# Impact of bovine respiratory disease on tissue-specific regulation of Zn and vitamin a metabolism and apparent absorption and retention of trace minerals

**DOI:** 10.1093/jas/skaf453

**Published:** 2025-12-31

**Authors:** Emma L Rients, Stephanie L Hansen, Jodi L Mcgill

**Affiliations:** Department of Animal Science, Iowa State University, Ames, IA 50011; Department of Animal Science, Iowa State University, Ames, IA 50011; Veterinary Microbiology and Preventive Medicine, Iowa State University, Ames, IA 50011

**Keywords:** bovine respiratory disease, trace minerals, vitamin A, cattle, nutritional immunity

## Abstract

This study aimed to characterize trace mineral and vitamin A metabolism and redistribution during clinical and subclinical respiratory infection in beef on dairy crossbred steers (n = 29; BW = 230 ± 2.14 kg). Steers were assigned to one of four groups encompassing days −6 to −1, 0 to 5, 5 to 10, and 10 to 15 of an experimental viral-bacterial respiratory challenge. Steers were adapted to metabolism crates for 5 d prior to a 5-d total urine and fecal collection period and necropsied at the end of the period. On day 0, steers were inoculated with bovine respiratory syncytial virus strain 375 followed by an intratracheal inoculation with *Mannheimia haemolytica* strain D153 on day 7. A natural disease challenge occurred during the study, leading to all steers showing signs of disease at necropsy. Lung pathology scores, plasma Fe concentrations, and rectal temperatures for 5 d prior to necropsy were used to categorize animals into clinical (n = 9) and subclinical (n = 20) disease. These categories were confirmed by decreases in dry matter intake (*P *= 0.06) and nitrogen retention (*P *= 0.06) in animals with clinical disease compared to subclinical. Plasma concentrations of Zn and retinol were lesser in clinical disease (*P *≤ 0.005). Conversely, liver (*P *= 0.02) and kidney (*P *= 0.06) concentrations of Zn were higher in clinical disease. This tissue sequestration occurred despite no difference in apparent Zn absorption or retention (*P *≥ 0.69), providing evidence of systemic mineral redistribution. There was also no difference in the apparent absorption of Cu, Fe, and Mn (*P *≥ 0.44), despite some differences in tissue concentrations. At the site of infection, expression of genes regulating vitamin A transport and metabolism (*STRA6*, *RXRα*, *RBP4*) increased (*P *≤ 0.002) in non-lesion lung relative to diseased lung. In both lesion and non-lesion lung, clinical disease decreased *RALDH2* expression relative to subclinical disease (*P *= 0.05). These findings demonstrate that BRD induces a coordinated redistribution of trace minerals from circulation to key tissues and alters local vitamin A metabolism in the lung. This highlights that plasma micronutrient concentrations during infection are not reflective of total body status, but rather an organized physiological response that prioritizes tissue-level demands.

## Teaser

Micronutrients are essential for the immune response; however, little is known about their metabolism during bovine respiratory disease infections. In this study, we characterize micronutrient metabolism during clinical and subclinical bovine respiratory disease challenges. This work provides a foundation for developing nutritional interventions to support cattle health during a disease of significant economic importance.

## Introduction

Bovine respiratory disease (BRD) is a leading cause of morbidity and mortality in the feedlot (NAHMS, 2013). Although BRD impacts all cattle, high risk populations such as beef on dairy crossbred cattle have heightened susceptibility, with one study reporting they were twice as likely to have BRD case fatality when compared to beef cattle (Theurer et al., 2020). Regardless of breed and background, understanding and improving nutrition of sick animals is critical to maintaining health and economic viability. Importantly, BRD triggers systemic inflammatory and metabolic responses that alter micronutrient utilization, suggesting that nutrient status may influence both disease progression and recovery.

Zinc and vitamin A are two micronutrients with critical and distinct roles in the immune response. Zinc is essential for immune cell proliferation (Shi et al., 1998) and function (Hasegawa et al., 2000). Vitamin A is essential for maintaining epithelial barrier integrity (Callaghan et al., 2020) and immune cell development (Mucida et al., 2007). Deficiency of vitamin A has been associated with increased risk of respiratory infections (Wang et al., 2021). Because both nutrients support epithelial defense, lymphocyte function, and coordinated inflammatory responses, and because their circulating concentrations decline during infection, they represent key micronutrients likely to be affected during BRD and relevant to evaluating host–pathogen interactions.

During BRD infections, nutrient metabolism shifts; this systemic change in micronutrient metabolism is known as nutritional immunity. In response to pathogen recognition, host cells produce proinflammatory cytokines, such as IL-6, which upregulate micronutrient transporters such as ZIP14 in the liver, which can increase the uptake of trace minerals such as Zn (Liuzzi et al., 2005), Mn (Fujishiro et al., 2014), and non-transferrin bound Fe (Liuzzi et al., 2006) resulting in decreased plasma concentrations of these minerals. A decrease in the circulating concentrations of Zn and Fe has been observed in beef cattle after lipopolysaccharide (LPS) injection (VanValin et al., 2021) and during BRD challenge (Wilson et al., 2016). Similarly, reductions in plasma retinol concentrations have been observed during immune activation, suggesting alterations in vitamin A metabolism in response to BRD (McGill et al., 2019). However, the extent to which these changes reflect altered absorption, tissue redistribution, or localized metabolic regulation remains unclear.

Despite the decrease in circulating micronutrients, adequate micronutrient status remains essential for the response to pathogens and recovery from infection. Severe vitamin A deficiency in young calves inhibited response to mucosal vaccine and BRSV disease challenge (McGill et al., 2019). In cattle of sufficient micronutrient status, administration of an injectable trace mineral containing Zn, Mn, Cu, and Se prior to or during BRD challenge resulted in improved disease resilience in weaned beef calves (Hong et al., 2025), suggesting increasing available micronutrients can improve the response to pathogens. Micronutrients are typically supplemented in the diet; however, changes in absorption and retention due to infection in cattle remain inadequately explored. Additionally, little is known about the concentrations of micronutrients in key tissues active in the immune response to BRD. Characterizing these dynamics is necessary to understand whether infection alters availability of key nutrients at sites where they support immune function.

Therefore, this study aimed to characterize trace mineral (Zn, Mn, Cu, and Fe) absorption and retention during a BRD challenge in beef on dairy crossbred steers. We also assessed trace mineral (Zn, Mn, Cu, and Fe) concentrations and expression of genes related to Zn and vitamin A metabolism in key immune and trace mineral homeostasis tissues to better understand their role in BRD response. We hypothesized increased disease severity would negatively affect trace mineral absorption and retention and alter gene expression related to Zn and vitamin A metabolism. This hypothesis is based on the rationale that a more severe infection elicits a stronger acute phase response, which can both impair nutrient absorption and drive greater cytokine-mediated redistribution and metabolic regulation of micronutrients within immune-responsive tissues.

## Materials and Methods

All protocols were approved by the institutional animal care and use committee (IACUC-21-003) and institutional biosafety committee (IBC-21-001).

### Animals and collections

**Figure 1.**
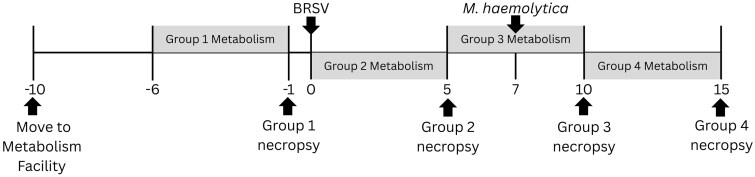
Timeline of study events. Steers were moved to the metabolism facility in Kildee Hall on day −10. On day 0, steers were inoculated with 10^4^ median tissue culture infectious dose (TCID_50_) BRSV 375 via nebulizer aerosol inoculation. On day 7 of viral challenge, all remaining steers received an intratracheal inoculation with 9.3 × 10^9^ colony forming units (CFU) *Mannheimia haemolytica* strain D153. Metabolism periods for groups 1, 2, 3, and 4 were days −6 to −1, 0 to 5, 5 to 7, and 10 to 15, respectively. At the end of each metabolism period, steers were humanely euthanized and necropsied.

Eight-week-old beef on dairy crossbred steers (n = 40; BW = 101 ± 0.71 kg) arrived at the Iowa State University Beef Nutrition farm (Ames, IA) and were managed as one group for three months prior to the disease challenge study. One week after arrival, all calves received Draxxin (tulathromycin; 2.5 mg/kg BW; Zoetis, Kalamazoo, MI) to mitigate a disease outbreak. Steers received chopped hay and a common pelleted diet meeting or exceeding NASEM (2016) recommendations for trace mineral and vitamin requirements. Twenty-nine days before viral challenge, steers were transitioned to a cottonseed hull pellet (CSHP) to replace chopped hay as a roughage source. A summary of experimental events is provided in Figure 1.

### Infection model

Ten days prior to viral infection, steers were weighed and 32 steers (BW =230 ± 2.14 kg) were chosen based on similar bodyweight (BW) and health status to be enrolled in the study. These were transported to Kildee Hall (6.8 km; Iowa State University, Ames, IA), where they were housed in the animal holding and metabolism facilities for the disease challenge study. Upon arrival, steers were in either group housing (4 steers/pen) or individual stainless-steel crates designed for total urine and feces collection (187.4 cm [length] × 76.2 cm [width] × 182.9 cm [height]). Group housed calves were bedded with wood chips while metabolism crates were equipped with rubber fatigue mats.

On d 0 steers were infected with 10^4^ median tissue culture infectious dose (TCID_50_) bovine respiratory syncytial virus (BRSV) 375 via nebulizer aerosol inoculation covering the nostrils and mouth as described by Sacco et al. (2012). The virus stock was isolated from diseased lung and passaged twice on primary bovine turbinate cells as described in McGill et al. (2018). Approximately 5 mL of the viral inoculum was delivered via forced-air nebulizer. Viral inoculation took approximately 5 min per steer, or until all viral inoculum was delivered.

On day 7 of viral challenge, all remaining steers received an intratracheal inoculation with 9.3 × 10^9^ Colony forming units (CFU) *Mannheimia haemolytica* strain D153 followed by 30 mL of saline. The bacterial culture was prepared using methods previously described by Hong et al. (2025). Briefly, pure cultures of *M. haemolytica* were plated on brain–heart infusion (BHI) agar plates 2 d prior to challenge. The morning of challenge, pure colonies were suspended in BHI broth and growth to log-phase in a 37°C shaking incubator. Bacterial cells were pelleted and washed several times with phosphate-buffered saline (PBS), then resuspended in sterile saline for inoculation. The infection dose was confirmed by quantitative culturing on BHI agar.

### Metabolism period

There were four groups allocated to periods of total urine and fecal collection and necropsy dates. Five days prior to the metabolism period, steers were moved into metabolism crates for adaptation. Group 1 (n = 8) received no experimental viral or bacterial challenge. They were adapted to crates days −10 to −7, total urine and fecal collections were day −6 through −1 and steers were necropsied on day −1. Groups 2–4 followed similar schedules, being adapted to crates for 4 d prior to a 5-d collection period and necropsy. Group 2 (n = 6) collection period was days 0–5 of viral challenge, group 3 (n = 8) collection was days 5–10 of viral challenge and group 4 (n = 6) was days 10–15 of viral challenge. During adaptation, feed offered each morning was 105% of the previous day’s intake. During the metabolism period, feed was limited to 95% of ad libitum intake observed during the adaptation period. All offered pellets and CSHP were recorded daily and intake was determined by subtracting the refused feed from the previous day’s offered feed. During the study, CSHP offered remained consistent at 1.35 kg/steer daily. During each period, a composite of CSHP and pellet was made. Samples of feed refused were collected daily and dried at 70°C for 48 h.

Methods for the preparation of metabolism crates as well as daily urine and fecal collections were followed as described in Pogge et al. (2014). Briefly, urine output was collected into 15–20 L carboys that were previously acid washed and contained 150 or 200 mL 6M acetic acid and 1 L of deionized water. There was greater urine output than expected, so urine output was monitored approximately every 12 h and collected if expected to be greater than carboy volume by the next 12 h checkpoint. Every carboy was sampled after 24 h of collection if not sampled before due to high urine output. Before removing a 1% aliquot of urine, urine pH was determined and additional acetic acid was included to achieve a pH of less than 5. Initial and added acetic acid and deionized water were recorded and subtracted from the total urine output prior to the aliquot calculation. The urine aliquot was composited into a 2-L container and frozen at −20°C. Total fecal output was collected on a tared piece of plastic sheeting. The fecal output and plastic sheeting were removed every morning of collection and plastic sheet was immediately replaced. Feces were weighed and a 3% aliquot was taken and dried at 70°C for 48 h.

All dried feeds, orts, and fecal samples were ground through a 2 mm screen (Retsch ZM 200; Verder Scientific Inc., Newtown, PA) and stored in an air-tight bag until further analysis. Fecal samples were composited by animal into one sample after grinding. True dry matter and organic matter (OM) were determined for feed, orts and fecal samples according to AOAC (2007). Briefly, 1.0 g of sample was weighed into a crucible and dried at 105°C for 24 h, the weight of the dry sample was recorded, then the dried sample was ashed in a muffle furnace (500°C for 12 h), and the weight of the ashed sample was recorded.

Dry matter of feed and fecal samples was calculated by multiplying the 105°C DM adjusted value of the feed or fecal sample for each steer. Dry matter intake (DMI) was determined by subtracting orts (DM basis) from feed offered (DM basis). Feed and fecal organic matter was determined by multiplying the percent OM of the feed or fecal sample by the 105°C DM adjusted value for that sample. Digestibility of both DM and OM was calculated by subtracting the DM or OM adjusted fecal output from the DM or OM adjusted total intake (pellets and CSHP minus orts), dividing the DM or OM adjusted total intake and subtracting the value from 100.

Urine was thawed at 4°C and composited into acid washed containers if more than one bottle was needed for storage. After stirring, urine was filtered using ashless filter paper (Cytiva Whatman541, Fisher scientific, Hampton, NH) to remove any additional contaminants. Urine was then aliquoted for nitrogen (N), mineral, and other analysis to limit freeze–thaw cycles.

### Clinical disease scoring

Steers were monitored daily for clinical disease by a single trained observer starting 10 d before viral infection. Steers were assigned a score as described in Mahmoud et al. (2020) . The scoring system assigns numbers (0–3) based on severity of clinical signs including (0 = no cough to 3= repeated spontaneous coughing), nasal discharge (0 = normal, serous discharge to 3 = copious bilateral mucopurulent nasal discharge), ocular discharge (0 = normal to 3 = heavy ocular discharge), and ear position (0 = normal to 3 = severe head tilt or bilateral ear droop). An additional category for respiratory effort was added (0 = no effort to 3 = significant effort). These scores were then added to determine the total clinical disease score for each steer daily.

In addition to clinical disease scores, rectal temperatures of steers in metabolism crates were taken daily and a score was assigned: 0 = < 38.3°C; 1 = 38.3–38.8°C; 2 = 38.9–39.3; 3 ≥ 39.4°C.

### Mineral and nitrogen analysis: feed, fecal, and urine

Feed and fecal samples were acid digested with 10 mL of nitric acid in a closed vessel microwave digestion system (CEM Mars Xpress; Matthews, NC) before mineral analysis according to Richter et al. (2012). Urine samples were diluted 7:10 with 3.3% nitric acid. Mineral analysis of plasma was determined using methods previously described (Pogge and Hansen, 2013). Tissues were dried at 70°C for 72 h prior to being acid digested with 5 mL of nitric acid in a closed vessel microwave digestion system. No additional dilution was used.

Mineral content was analyzed using inductively coupled plasma optical emission spectrometry (ICP-OES; Optima 7000; PerkinElmer, Walthman, MA). Quality control samples (serum UTAK, Valencia, Ca; bovine liver form National Institutes of Standards and Technology, Gaithersburg, MD) were included with each run to verify instrument accuracy. An internal standard of yttrium (PerkinElmer) was added to all samples to account for variation in sample introduction. Feed, fecal, and urine samples were analyzed in duplicate for nitrogen (AOAC, 2007; Trumac LECO Corp., St Joeseph, MI).

Calculations of total mineral and nitrogen concentration of each component (feed offered and refused, feces, and urine), retention and apparent absorption were completed using the methods described by Pogge et al. (2014).

### Blood collection

Jugular blood was collected from steers in trace mineral grade potassium EDTA and serum vacuum tubes (Becton, Dickinson and Company, Franklin Lakes, NJ). For group 1, blood was collected on day of necropsy. For all other steers, blood was collected on days 0, 5, 7, 10, and 15 of viral infection. Samples were centrifuged at 1,000×*g* for 20 min at 4°C. Plasma for trace mineral analysis was stored at −20°C until analysis. Plasma for vitamin A analysis and serum was stored a −80°C until analysis.

### Necropsy

Steers were transported to the Iowa State University College of Veterinary Medicine (3.9 km) and humanely euthanized by barbiturate overdose. Pathological evaluation was performed similar to previous descriptions (Sacco et al., 2012). Lungs were graded on pneumonic consolidation using a previously published scoring system (McGill et al., 2018). A score was assigned based on the percentage of lung affected by gross pneumonic lesions (6 = free of lesions; 5 = 1% to 5% affected; 4 = 6% to 15%; 3 = 16% to 30%; 2 = 31% to 50%; 1 = >50%). Examples of lung scores are included in Supplementary Figure S1. Lungs were rinsed with saline prior to tissue sampling to collect bronchoalveolar lavage fluid and cells.

Several tissues were sampled and stored for further analysis. Prior to storage, samples were rinsed thoroughly with PBS. Samples for mineral concentrations (lesion and non-lesion lung, liver, kidney, spleen, tracheobronchial lymph node [LN], pancreas, and thymus) were stored in whirlpack bags at −20°C until analysis. Additional tissues (lesion [LL] and non-lesion [NLL] lung, liver, kidney, tracheobronchial LN and mesenteric LN and jejunum scraping) were stored in RNA*later* (Invitrogen, Life Technologies) at −80°C until analysis. Another subset of liver tissue was snap frozen in liquid N and stored at −80°C for analysis.

### Vitamin A analysis

Plasma vitamin A (or serum for group 1) was determined using the iCheck Flouro (BioAnalyt, Telto, Germany) according to manufacturer’s instructions. Liver vitamin A was determined using the same system with additional preparation of sample. First, 0.1 to 0.2 g liver was weighed and added to a 15 mL conical tube. Ten mL of ice cold deionized water was immediately added and the sample was homogenized (Polytron, Brinkmann Instruments, Woodbury, NY). The sample was then immediately drawn up and added to an iCheck vial for analysis via manufacturer’s instructions.

### Mineral analysis: tissues and plasma

A portion of the tissues stored at −20°C were dried at 70°C in a forced-air oven for at least 72 h. The dry matter of these tissues is reported in Supplementary Table S1. Approximately 0.3 g of sample was digested in 5 mL of nitric acid in a closed vessel microwave digestion system using methods previously described (Pogge and Hansen, 2013). All tissues except for liver were digested and analyzed in duplicate. Plasma samples were diluted 1:8 in trace-mineral grade nitric acid and prepared as previously described (Pogge and Hansen, 2013). Samples were analyzed using ICP-OES as described above.

### Gene expression

Total RNA was isolated from tissue samples and complimentary deoxyribonucleic acid (cDNA) was prepared as previously described in McGill et al. (2016). Briefly, 0.03 g of tissue was homogenized with 1 mL of TRIzol (Invitrogen, Waltham MA). Chloroform (200 µL) was added and samples were vortexed, incubated for 3 minutes at room temperature and then centrifuged at 18,500×*g* for 15 min. Following centrifugation, the upper, clear layer was removed and diluted with equal volume of 70% ethanol. The extraction mixture was added to a RNeasy Mini spin column and washed using buffers supplied in the RNeasy RNA isolation kit (Qiagen, Hilden, Germany). Additionally, RNA was treated with using the RNase-free DNase set (Qiagen) for 20 min at room temperature (Qiagen). The isolated RNA was eluted with RNAse-free water and the Qubit RNA broad range kit (Thermo Fisher Scientific, Waltham, MA) was used to determine RNA concentration using a Qubit 4 fluorometer (Invitrogen). For cDNA synthesis, 500 ng of RNA was added to a master mix containing 100 ng of random primers (Invitrogen) and 20 nM of dNTP (Invitrogen) and incubated at 70°C for 5 min. Then a master mix containing 5X first strand buffer (Invitrogen), 0.1 DTT (Invitrogen), RNAse Out (Invitrogen) and SuperScript III (Invitrogen) was added. Samples were incubated at 25°C for 5 min, followed by an incubation at 50°C for 1 h and 80°C for 5 min. All cDNA incubations were completed using the MiniAmp Thermocycler (Applied Biosystems, Waltham, MA). Before qPCR, cDNA was diluted 1 to 10 with RNAse-free water. The qPCR reactions were performed using SYBR Green Power PCR Mastermix (Thermo Fisher Scientific) using a QuantStudio 5 qPCR machine (Applied Biosystems) as previously reported (McGill et al., 2016). Relative gene expression is expressed as a delta cycle threshold (ΔCT) and was determined by subtracting the housekeeping gene (RPS9) from the gene of interest (Hong et al., 2025). The primer sets are listed in Supplementary Table S2.

### Illness groupings and statistical analysis

A natural respiratory disease outbreak occurred during the study, causing all steers, including non-challenged steers, to exhibit clinical signs of illness and lung lesions indicating respiratory illness. Therefore, a degree of illness scale was developed utilizing the average rectal temperature for the 5 d prior to necropsy, plasma Fe on day of necropsy, and lung lesion score at necropsy to categorize steers into clinical and subclinical illness groups. Fever is a non-specific indicator of disease, and both BRSV and *M. haemolytica* challenges result in increased rectal temperatures after inoculation (Grissett et al., 2015). The rectal temperature of steers in this study was collected daily and converted into a score (0 = < 38.3°C; 1 = 38.3-38.8°C; 2 = 38.9-39.3; 3 ≥ 39.4°C). All steers had these scores recorded for the 5 d prior to necropsy, and the average of these scores was used for groupings.

Plasma Fe concentrations were included in the groupings due to the well-described decrease in circulating Fe caused by proinflammatory stimuli. This decrease is due to upregulation of hepcidin and ZIP14, inhibiting the release of Fe into circulation and increasing Fe uptake into tissue for storage (Nemeth et al., 2003; Liuzzi et al., 2006). This decrease in plasma Fe has previously been observed in BVDV and *M. haemolytica* coinfection (Wilson et al., 2016). Plasma Fe was assigned a value of 1 if less than 0.7 mg/L and 2 if greater than 0.7 mg/L.

Lastly, lung pathology scores, discussed above, were utilized in grouping due to their indication of respiratory disease, with previous studies utilizing this measure at harvest to understand impacts of BRD on growth performance and carcass characteristics (Rezac et al., 2014).

The degree of illness score was calculated by adding the average rectal temperature, plasma Fe and lung pathology scores. The degree of illness score allows us to group steers according to disease state at time of metabolism and necropsy, regardless of time surrounding infection. If the sum of these variables was less than 10, they are considered to have clinical disease (n = 9). If the sum of variables is greater than 10, they are considered to have subclinical disease (n = 20). Average concentrations and scores for these variables are presented in Table 1. One steer in the clinical disease group was necropsied prior to metabolism collection and was included in the dataset for all other variables.

**.able 1.** Comparison of disease score measures between clinical and subclinical disease

	Clinical^1^	Subclinical^1^	SEM	*P* value
**n**	9	20		
**Plasma Fe**	0.76	1.47	0.180	0.0007
**Rectal temperature score^2^**	2.0	1.3	0.11	0.001
**Lung pathology score^3^**	2.6	3.7	0.36	0.01
**Clinical score^4^**	2.9	1.2	0.35	0.0004

1 Steers were allocated into clinical and subclinical groups based on plasma Fe concentrations, lung pathology scores, and rectal temperatures during metabolism collection.

2 Rectal temperatures were taken daily. Temperatures were assigned a score: 0 ≤ 38.3°C; 1 = 38.3–38.8°C; 2 = 38.9–39.3°C; 3 ≥ 39.4°C. Scores for the 5 d prior to necropsy were averaged.

3 At necropsy, lungs were scored based on the percentage of lung affected by gross pneumonic lesions: 6 = 0% affected; 5 = 1% to 5% affected; 4 = 6% to 15%; 3 = 16% to 30%; 2 = 31% to 50%; 1 = >50%.

4 Calves were monitored daily for clinical signs of illness and scored using an adaption of the University of Wisconsin Calf Health Respiratory Scoring chart. Scores for the 5 d prior to necropsy were averaged.

Data were analyzed using the Mixed procedure of SAS 9.4 (Cary, NC). The model included the fixed effect of illness group. For lung data, a multivariate paired analysis was used to compare the effects of tissue type (lesion or non-lesion), illness group and their interaction. Covariance structures were tested for best model fit using AICC and compound symmetry was utilized for analysis. Model fit and assumptions were evaluated by examining the residuals. Outliers were defined as data points greater than three standard deviations from the treatment mean, based on residual and were removed. Statistical significance is defined as *P *≤ 0.05 and a tendency 0.1 ≥ *P *> 0.05.

## Results

### DM, OM, and N digestibility and retention

Clinically diseased steers tended to have decreased DM and N intake (Table 2; *P *≤ 0.08) and had decreased OM intake (*P *= 0.05) and fecal output (*P *= 0.004), but urine output did not differ from subclinical disease (*P *= 0.73). Fecal OM and N were decreased in clinical disease (*P *≤ 0.01), but OM and N digestibility were not different between groups (*P *≥ 0.46). Urinary N excretion was not different between groups (*P *= 0.20). Nitrogen retention (g/day and %) tended to be decreased in clinical disease (*P *≤ 0.06).

**Table 2.** Effects of bovine respiratory disease challenge on daily dry matter intake, diet digestibility, nitrogen digestibility, and daily urine and fecal output

	Clinical^1^	Subclinical^1^	SEM	*P* value
**Steers (n)**	8	20		
**DMI, kg/d**	4.5	5.5	0.43	0.06
**OM intake, kg/d**	4.2	5.2	0.40	0.05
**N intake, g/d**	127.1	159.5	14.76	0.08
**Daily output**				
**Fecal, kg DM/d**	1.1	1.5	0.10	0.004
**Fecal OM, kg/day**	0.9	1.4	0.09	0.001
**Fecal N, g/day**	28.3	36.8	2.42	0.01
**Urine, L/d**	12.3	13.1	1.98	0.73
**Urine N, g/day**	46.8	57.6	7.05	0.20
**Digestibility**				
**DM digestibility, %**	74.1	72.9	1.31	0.46
**OM digestibility, %**	74.1	72.9	1.31	0.46
**N digestibility, %**	75.6	76.3	1.90	0.74
**N retention**				
**N retention, g/day**	42.1	65.15	10.00	0.06
**N retention, %**	27.6	40.24	5.38	0.06

1 Steers were allocated into clinical and subclinical groups based on plasma Fe concentrations, lung pathology scores, and rectal temperatures during metabolism collection.

### Zn, Cu, Fe, and Mn digestibility and retention

Intake of Zn and Cu was lesser in clinical disease (Table 3; *P *≤ 0.05) while Mn intake tended to be lesser (*P *= 0.06) and Fe intake did not differ (*P *= 0.13). Fecal excretion (mg/day) of Zn, Fe, and Mn was decreased in clinical disease (*P *≤ 0.05) while fecal Cu excretion tended to be lesser (*P *= 0.06). Fecal excretion as a percent of intake was not different between groups for Zn, Cu, Fe, and Mn (Table 4; *P *≥ 0.41). Urinary excretion (mg/day and percent of intake) of Zn, Cu, Fe, and Mn was not different between groups (*P *≥ 0.17). Retention (mg/day and as percent of intake) of Zn, Cu, Fe, and Mn was not different between groups (*P *≥ 0.21). Apparent absorption of Zn, Cu, Fe, and Mn was not different between groups (*P *≥ 0.41).

**Table 3.** Effects of bovine respiratory disease challenge on the amount (mg/d) of daily micromineral intake, fecal and urine excretion, mineral retention, and mineral retention as a percent of intake

	Clinical^1^	Subclinical^1^	SEM	*P* value
**Steers (n)**	8	20		
**Mineral intake**				
**Zn**	273	347	29.0	0.04
**Cu**	62	81	7.5	0.05
**Fe**	602	691	59.2	0.20
**Mn**	223	279	23.7	0.06
**Fecal excretion**				
**Zn**	233	299	26.4	0.04
**Cu**	55	69	6.4	0.06
**Fe**	619	743	54.9	0.06
**Mn**	223	264	16.5	0.05
**Urinary excretion**				
**Zn**	0.98	1.27	0.203	0.24
**Cu**	0.18	0.24	0.038	0.17
**Fe**	0.78	1.00	0.145	0.20
**Mn**	0.32	0.35	0.075	0.72
**Mineral retention**				
**Zn**	39	46	14.0	0.69
**Cu**	7	11	2.6	0.21
**Fe**	−19	−53	47.9	0.53
**Mn**	−1	15	19.7	0.50

1 Steers were allocated into clinical and subclinical groups based on plasma Fe concentrations, lung pathology scores and rectal temperatures during metabolism collection.

**Table 4.** Effects of bovine respiratory disease challenge on, fecal and urine excretion, mineral retention, mineral retention, and mineral absorption as a percent of intake

	Clinical^1^	Subclinical^1^	SEM	*P* value
**Steers (n)**	9	20		
**Fecal excretion**				
**Zn**	86.2	86.5	3.97	0.95
**Cu**	85.6	86.3	3.22	0.85
**Fe**	108.8	109.2	8.11	0.97
**Mn**	98.9	96.5	7.82	0.80
**Urinary excretion**				
**Zn**	0.38	0.37	0.063	0.89
**Cu**	0.30	0.29	0.040	0.83
**Fe**	0.17	0.15	0.025	0.38
**Mn**	0.14	0.12	0.026	0.44
**Mineral retention**				
**Zn**	13.4	13.1	3.95	0.95
**Cu**	14.1	13.5	3.21	0.85
**Fe**	−7.7	−9.3	7.47	0.86
**Mn**	1.0	3.3	7.82	0.80
**Apparent absorption**				
**Zn**	13.8	13.5	3.97	0.95
**Cu**	10.3	13.7	3.65	0.44
**Fe**	−8.8	−9.2	8.11	0.97
**Mn**	1.1	3.5	7.82	0.80

1 Steers were allocated into clinical and subclinical groups based on plasma Fe concentrations, lung pathology scores, and rectal temperatures during metabolism collection.

### Tissue and plasma micronutrient concentrations

Liver concentrations of Fe and Zn were greater in clinical disease (Table 5; *P *≤ 0.02). There was no difference in the liver concentration of Cu, Mn, and retinol (*P *≥ 0.24). Kidney concentrations of Zn tended to be greater in clinical disease (*P *≥ 0.06). There were no differences in kidney concentrations of, Cu, Fe, and Mn (*P *≥ 0.36). No differences in the thymus concentrations Cu, Fe, Mn, or Zn were noted (*P *≥ 0.11). In the tracheobronchial LN, concentrations of Cu, Fe, and Mn were greater in clinical disease (*P *≤ 0.02) while Zn concentrations tended to be lesser (*P *≤ 0.09) There was greater Cu in the spleen in clinical disease (*P *= 0.04). There were no differences in spleen, Fe, Mn, and Zn concentrations (*P *≥ 0.18). In the pancreas, Mn was lesser in clinical disease (*P *< 0.01). There were no differences in the concentrations of Cu, Fe, and Zn in the pancreas (*P *≥ 0.24).

**Table 5.** Effects of bovine respiratory disease challenge on micronutrient concentration (mg/kg DM) of liver, thymus, kidney, spleen, tracheobronchial lymph node, and pancreas

	Clinical^1^	Subclinical^1^	SEM	*P* value
**Steers (n)**	9	20		
**Liver**				
**Zn**	307	213	31.7	0.02
**Cu**	422	468	32.1	0.24
**Fe**	304	198	27.3	0.003
**Mn**	11.0	11.3	0.61	0.62
**Retinol**	255	252	20.8	0.89
**Thymus**				
**Zn**	53	63	5.5	0.12
**Cu**	1.6	1.1	0.32	0.21
**Fe**	47	42	4.0	0.31
**Mn**	0.5	0.5	0.07	0.91
**Kidney**				
**Zn**	104	90	5.8	0.06
**Cu**	18.3	17.8	0.93	0.67
**Fe**	188	174	12.1	0.36
**Mn**	3.98	3.85	0.265	0.69
**Spleen**				
**Zn**	55	50	4.5	0.35
**Cu**	3.7	3.4	0.12	0.04
**Fe**	2103	2042	145.8	0.73
**Mn**	0.52	0.44	0.048	0.18
**Tracheobronchial lymph node**				
**Zn**	77	81	1.8	0.06
**Cu**	4.08	3.56	0.166	0.02
**Fe**	137	83	11.8	0.001
**Mn**	1.50	1.15	0.117	0.02
**Pancreas**				
**Zn**	127	134	10.7	0.57
**Cu**	3.5	3.7	0.10	0.24
**Fe**	71	67	6.4	0.70
**Mn**	3.66	4.95	0.249	0.0002

1 Steers were allocated into clinical and subclinical groups based on plasma Fe concentrations, lung pathology scores, and rectal temperatures during metabolism collection.

**Figure 2.**
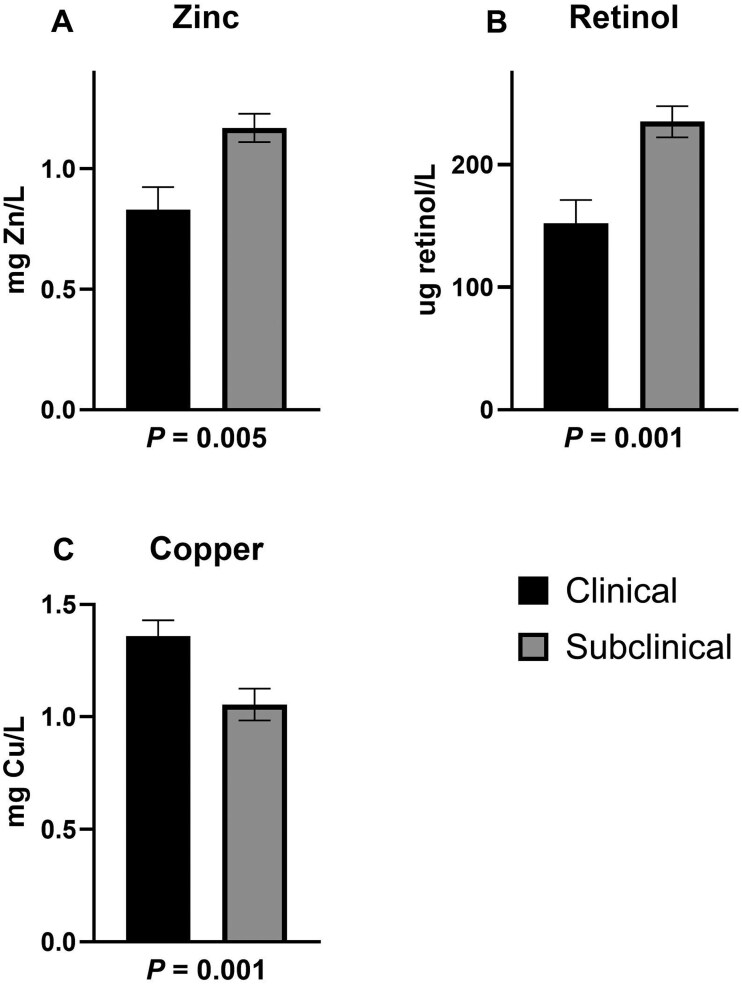
Effects of clinical and subclinical bovine respiratory disease challenge on plasma concentrations on day of necropsy. Steers were allocated into clinical and subclinical groups based on plasma Fe concentrations, lung pathology scores, and rectal temperatures during the five days prior to necropsy. (A) Plasma Zn concentrations were lesser in clinically diseased steers (*P *= 0.005). (B) Plasma retinol concentrations were lesser in clinically diseased steers (*P *= 0.001). (C) Plasma Cu concentrations were greater in clinically diseased steers (*P *= 0.001).

On day of necropsy, plasma Zn and retinol concentrations were lesser in clinical disease (Figure 2; *P *< 0.01). Plasma Cu concentrations were greater in clinical disease (*P *< 0.01).

There was an interaction between group and tissue type for Cu concentrations in lesion and non-lesion lung (Table 6; *P *= 0.03) where clinical LL is greater than NLL and while LL and NLL in subclinical disease are intermediate. There was an interaction between group and tissue for Zn concentrations in lesion and non-lesion lung (*P *= 0.03) where clinical disease LL and subclinical disease LL were lesser in Zn than subclinical disease NLL.

**Table 6.** Effects on tissue lesion and bovine respiratory disease challenge on lung mineral concentrations (mg/kg DM)

	Clinical^1^		Subclinical^1^		SEM	Group × Tissue *P* value	Group *P* value	Tissue *P* value
	**LL^2^**	**NLL^2^**	**LL^2^**	**NLL^2^**				
**Zn**	78.8^a,b^	74.3^a,b^	81.0^a,b^	87.2^a,b^	3.67	0.03	0.06	0.72
**Cu**	5.5^a,b^	4.5^a,b^	4.8^a,b^	4.9^a,b^	0.331	0.03	0.70	0.06
**Fe**	426.2	502.5	301.5	436.0	41.80	0.23	0.04	<0.01
**Mn**	0.70	0.42	0.72	0.50	0.063	0.63	0.36	<0.01

1 Steers were allocated into clinical and subclinical groups based on plasma Fe concentrations, lung pathology scores, and rectal temperatures during metabolism collection; Clinical n = 9; subclinical n = 20.

2 LL, lesion lung; NLL, non-lesion lung.

^a,b^Within row, means with different superscripts differ P ≤ 0.05

There were no interactions between group and tissue for Fe and Mn concentrations (*P *≥ 0.23). Concentrations of Fe were greater in clinical disease (group *P *= 0.04) and Fe concentrations were greater in LL compared to NLL (tissue *P *< 0.01). Concentrations of Mn were greater in LL compared to NLL (tissue *<* 0.01) and were not different between groups (group *P *= 0.36).

### Liver gene expression

In the liver, gene expression of *HAMP* was increased in clinical disease (Table 7; *P *= 0.05). Subclinical disease increased gene expression of *AHR*, *RALDH2*, *RBP1*, *ZNT1*, *ZNT4*, and *ZIP8* (Tables 7 and 8; *P *≤ 0.05) in the liver. There was no difference in the expression of *RXRα*, *SAA1*, *RBP4*, *ZIP1*, *ZIP2*, and *ZIP14* (*P *≥ 0.11).

**Table 7.** Effects of bovine respiratory disease challenge on gene expression (dCT^1^) of mineral metabolism related genes in liver, kidney, jejunum, mesenteric lymph node, and draining lymph node

	Clinical^2^	Subclinical^2^	SEM	*P* value
**Steers (n)**	9	20		
**Liver**				
**ZIP1**	3.24	3.01	0.151	0.21
**ZIP2**	9.89	9.15	0.468	0.19
**ZIP8**	3.54	2.54	0.301	0.01
**ZIP14**	1.39	1.00	0.306	0.30
**ZnT1**	1.47	0.97	0.195	0.05
**ZnT4**	5.94	5.45	0.149	0.01
**HAMP**	−2.07	−0.81	0.509	0.05
**Kidney**				
**ZIP1**	3.70	3.92	0.218	0.41
**ZIP2**	9.08	8.13	0.571	0.18
**ZIP8**	2.98	3.89	0.329	0.03
**ZIP14**	7.13	7.17	0.559	0.95
**ZnT1**	4.12	3.88	0.397	0.63
**Znt4**	6.25	6.34	0.206	0.74
**Jejunum**				
**ZIP4**	4.38	4.99	0.699	0.47
**ZIP8**	7.19	7.98	0.489	0.20
**ZnT1**	2.39	3.15	0.260	0.02
**S100A8**	9.40	10.30	0.882	0.41
**Mesenteric lymph node**				
**ZIP1**	5.25	5.24	0.144	0.97
**ZIP2**	13.27	12.65	0.391	0.20
**ZIP8**	10.54	10.57	0.241	0.91
**ZIP14**	7.61	7.44	0.249	0.58
**ZnT1**	4.00	3.87	0.247	0.67
**ZnT4**	7.37	7.32	0.173	0.79
**Tracheobronchial lymph node**				
**ZIP1**	5.07	4.94	0.183	0.57
**ZIP2**	12.17	12.24	0.498	0.90
**ZIP8**	8.28	8.58	0.505	0.62
**ZIP14**	6.83	7.25	0.252	0.16
**ZnT1**	3.86	3.79	0.143	0.70
**ZnT4**	7.04	6.82	0.203	0.37

1 dCT calculated using RPS9 as reference gene.

2 Steers were allocated into clinical and subclinical groups based on plasma Fe concentrations, lung pathology scores, and rectal temperatures during metabolism collection.

**Table 8.** Effects of bovine respiratory disease challenge on gene expression (dCT^1^) of vitamin A metabolism related genes in liver, kidney, jejunum, mesenteric lymph node, and draining lymph node

	Clinical^2^	Subclinical^2^	SEM	*P* value
**Steers (n)**	9	20		
**Liver**				
**AHR**	3.81	2.71	0.304	0.006
**RALDH2**	9.44	8.07	0.431	0.01
**RBP1**	7.40	6.37	0.315	0.01
**RBP4**	0.27	−0.72	0.508	0.12
**RXRα**	1.30	1.33	0.175	0.89
**Kidney**				
**RALDH2**	11.23	10.44	0.678	0.32
**RBP1**	6.11	5.58	0.367	0.25
**RBP4**	8.13	8.12	0.319	0.99
**STRA6**	2.47	3.44	0.345	0.03
**Jejunum**				
**BCMO1**	3.39	2.85	1.281	0.73
**MUC5AC**	10.30	10.56	0.904	0.81
**RALDH2**	10.00	9.59	0.464	0.47
**RBP1**	9.54	9.98	0.720	0.61
**RBP4**	10.57	11.44	0.667	0.29
**RXRα**	3.35	3.99	0.218	0.02
**STRA6**	9.38	9.25	0.703	0.88
**OCLN**	3.38	3.93	0.281	0.11
**Mesenteric lymph node**				
**RALDH2**	14.36	13.55	0.859	0.44
**RBP1**	8.47	8.15	0.314	0.40
**RBP4**	10.39	10.40	0.361	0.99
**RXRα**	5.30	5.62	0.274	0.33
**STRA6**	7.76	7.25	0.160	0.01
**Tracheobronchial lymph node**				
**RBP1**	8.17	8.18	0.347	0.98
**RBP4**	8.87	8.73	0.478	0.81
**RXRα**	5.19	5.32	0.128	0.39
**STRA6**	9.07	8.54	0.318	0.16

1 dCT calculated using RPS9 as reference gene.

2 Steers were allocated into clinical and subclinical groups based on plasma Fe concentrations, lung pathology scores, and rectal temperatures during metabolism collection.

### Kidney gene expression

Clinical disease increased gene expression of *ZIP8* and *STRA6* in the kidney (Tables 7 and 8; *P *= 0.03). There were no differences in the expression of *RALDH2*, *RBP1*, *RBP4*, *ZIP1*, *ZIP2*, *ZIP14*, *ZNT1*, and *ZNT4* in the kidney (*P *≥ 0.18).

### Lung gene expression

There was an interaction between tissue and group for gene expression of *OCLN* in lungs (Table 9; group × tissue *P *= 0.03) where subclinical NLL had the greatest expression followed by clinical NLL, subclinical LL, and clinical LL. There was a tendency for an interaction for gene expression of *ZIP8* (group × tissue *P *= 0.10) where clinical LL was increased compared to subclinical LL, clinical and subclinical NLL. There were no other interactions between group and tissue for lung gene expression (group × tissue *P *≥ 0.11).

**Table 9.** Effects on tissue lesion and bovine respiratory disease challenge on lung gene expression (dCT^1^)

	Clinical^2^		Subclinical^2^		SEM	Group × tissue *P* value	Group *P* value	Tissue *P* value
	**LL**	**NLL**	**LL**	**NLL**				
**AHR**	2.89	2.53	1.87	1.98	0.176	0.11	0.0001	0.38
**MUC5AC**	8.78	10.48	8.42	10.60	0.980	0.78	0.88	0.03
**MMP9**	6.45	9.24	7.79	10.40	0.675	0.88	0.03	0.0002
**OCLN**	3.62^a^	1.55^c^	2.16^b^	1.00^d^	0.246	0.03	0.0001	0.0001
**RALDH2**	10.00	11.68	8.85	10.37	0.684	0.89	0.05	0.01
**RBP1**	8.33	8.61	8.33	8.31	0.372	0.63	0.63	0.66
**RBP4**	6.78	5.20	6.90	5.67	0.324	0.56	0.24	0.0001
**RXRα**	3.26	2.19	3.30	2.57	0.176	0.30	0.13	0.0001
**STRA6**	7.94	9.31	7.91	9.77	0.559	0.62	0.65	0.002
**Zip1**	3.20	2.49	2.83	2.26	0.177	0.66	0.06	0.0003
**Zip2**	6.77	6.03	6.47	5.52	0.337	0.68	0.18	0.005
**Zip8**	2.66	3.76	3.75	4.10	0.279	0.10	0.01	0.003
**Znt1**	2.04	0.76	1.50	0.61	0.206	0.28	0.04	0.0001
**Znt4**	6.34	5.91	6.10	5.46	0.215	0.58	0.05	0.01

1 dCT calculated using RPS9 as reference gene.

2 Steers were allocated into clinical (n = 9) and subclinical (n = 20) groups based on plasma Fe concentrations, lung pathology scores, and rectal temperatures during metabolism collection.

a,b,c,d Within row, means with different superscripts differ *P *≤ 0.05.

Expression of *ZIP1*, *ZIP2*, *ZNT1*, and *ZNT4* was increased in NLL compared to LL (tissue *P *≤ 0.01). Gene expression of *ZNT1* and *ZNT4* was increased in subclinical disease (group *P *≤ 0.05). There was a tendency for increased gene expression of *ZIP1* in subclinical disease (group *P *= 0.06). There was no difference in expression of *ZIP2* between groups (group *P *= 0.18).

Expression of *RBP4*, *RXRα*, and *STRA6* was increased in NLL (tissue *P *< 0.01). Gene expression of *RALDH2* was increased in clinical disease (group *P *= 0.05). Expression of *RALDH2* was also increased in LL compared to NLL (tissue *P *= 0.05). There was no difference between the expression of *RBP4*, *RXRα*, and *STRA6* between groups (group *P *≥ 0.13). There was no difference between group and tissue for expression of *RBP1* (*P *≤ 0.66). Gene expression of *AHR* was greater in subclinical disease (group *P *< 0.01) and was not different between tissues (tissue *P *= 0.38).

Expression of *MUC5AC* was increased in LL compared to NLL (tissue *P *= 0.03) but was not different between groups (group *P *= 0.88). Expression of *MMP9* was increased in LL compared to NLL (tissue *P *= 0.0002) and increased in clinical disease (group *P *= 0.03).

### Lymph node gene expression

Gene expression of *Stra6* was increased in subclinical disease (*P *= 0.01) in mesenteric LN. There were no differences between groups in the gene expression of *RALDH2*, *RBP1*, *RBP4*, *RXRα*, *ZIP1*, *ZIP2*, *ZIP8*, *ZIP14*, *ZNT1*, and *ZNT4* (Tables 7 and 8; *P *≥ 0.20) in mesenteric LN. Gene expression for *RBP1*, *RBP4*, *RXRα*, *STRA6*, *ZIP1*, *ZIP2*, *ZIP8*, *ZIP14*, *ZNT1*, and *ZNT4* was not different between groups in tracheobronchial LN (*P *≥ 0.15).

### Jejunum gene expression

Gene expression of *RXRα* and *ZNT1* was increased in clinical disease (Tables 7and 8; *P *≤ 0.02) in jejunum. There were no differences in the gene expression of *BCMO1*, *MUC5AC*, *OCLN*, *RALDH2*, *RBP1*, *RBP4*, *S100A8*, *STRA6*, *ZIP4*, and *ZIP8* (*P *≥ 0.11) in jejunum.

## Discussion

Bovine respiratory disease is a major contributor to morbidity and mortality in the dairy, and beef industries. In this study we utilized plasma Fe concentrations, fever scores and lung pathology scores to assign steers to either clinical or subclinical BRD. All animals were exposed to an infection and had lung pathology scores, confirming respiratory disease, but clinical animals exhibited more severe illness and would be candidates for treatment based on the well accepted depression, appetite, respiratory, and temperature (DART) clinical scoring system (Griffin, 2010). These findings were further validated by an 18% decrease in DMI and reduced nitrogen retention (31% lower) in clinical steers compared to subclinical steers, consistent with previous reports of decreased intake and increased nitrogen excretion during experimental respiratory infections (Cole et al., 1986; Wilson et al., 2016; Broadway et al., 2021). Using these validated groupings, we compared the apparent absorption and retention of trace minerals, concentrations of trace minerals, and gene expression related to Zn and vitamin A in key metabolically active tissues during clinical and subclinical BRD.

**Figure 3.**
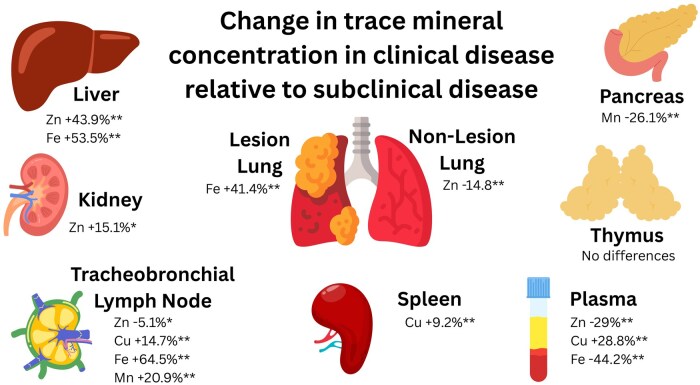
Change in trace mineral concentrations in tissues from steers in clinical disease relative to subclinical disease. Steers were allocated into clinical and subclinical groups based on plasma Fe concentrations, lung pathology scores, and rectal temperatures during the five days prior to necropsy. Differences between groups *P *≤ 0.05 indicated by **; 0.05 < *P *≤ 0.10 indicated by *.

Zinc is a key micronutrient in immune regulation, and its distribution shifted during BRD (Figure 3). In human macrophages, stimulation with LPS induces ZIP8 expression and when stimulated with Zn and LPS, there is reduced production of IL-10, which serves as negative feedback for cytokines like IL-6 (Pyle et al., 2017). Under ZIP8 knockout conditions, IL-10 is not affected (Pyle et al., 2017), indicating ZIP8 can mediate inflammatory signaling after bacterial stimulation. Similar roles of Zn and ZIP8 may also influence the inflammatory environment in the lung during infection in cattle. In the lung, clinically ill calves had higher *ZIP8* expression in LL, while broader transporter differences resulted in greater Zn concentrations in subclinical NLL compared to clinical NLL or LL. Although numerically small, these shifts could still have implications in lung immunity, given the tight homeostatic regulation of Zn.

In the current study, clinical steers decreased plasma Zn concentrations by 29% compared to subclinical steers on day of necropsy. In response to proinflammatory stimuli and pathogens, Zn transporters are upregulated (Liuzzi et al., 2005; Besecker et al., 2008), causing a reduction in circulating Zn concentrations. In the current study, *ZIP8* expression was increased in the kidney tissue of clinically ill animals. Kidney Zn concentrations also tended to be increased by 15% in clinical animals compared to subclinical, although urinary Zn excretion did not differ. This implies Zn may be stored in the kidney during the disease and later released after disease subsides. Interestingly, disease severity did not affect overall Zn apparent absorption or retention, but clinical cattle showed increased jejunal gene expression of *ZNT1*, the transporter responsible for moving Zn from enterocyte into circulation. While ZNT1 is typically upregulated by high intracellular Zn concentrations in non-diseased states (Nishito and Kambe, 2019) it is unclear whether this response in our cattle reflects plasma Zn status or disease state.

Previous literature suggests the liver as the main organ of Zn sequestration during infection and inflammation (Aydemir et al., 2012). In this study, steers with clinical disease had a 44% increase in hepatic Zn, confirming it is a location of Zn sequestration in cattle during disease. This was accompanied by reduced expression of *ZNT1* and *ZNT4* suggesting decreased mobilization during clinical disease, while Z*IP14* did not differ between groups. In contrast, tracheobronchial LN from clinical animals had lower Zn concentrations, possibly due to a dilution during LN hypertrophy (McLachlan et al., 2003), while spleen, thymus, and pancreas were unaffected. Overall, while Zn absorption and retention did not change, plasma, lung, liver, kidney, and tracheobronchial LN were key sites of Zn redistribution during BRD.

On day of necropsy, clinically infected animals had decreased plasma retinol concentrations by 36% compared to subclinical disease. Similar decreases in circulating vitamin A and retinol binding protein (RBP) concentrations have been observed in hospitalized respiratory syncytial virus (RSV) patients (Quinlan and Hayani, 1996). This decrease may reflect reduced mobilization of hepatic stores (Gieng et al., 2007). In the current study, liver vitamin A concentrations did not differ at necropsy; however, baseline values were unknown. Clinical disease, however, caused upregulation of *RBP1*, *retinaldehyde dehydrogenase 2* (RALDH2) and *aryl hydrocarbon receptor* (AHR) in the liver, suggesting greater local utilization rather than mobilization of vitamin A from the liver. Clinically infected animals had greater jejunal expression of *retinoid X receptor* (*RXR*) *α*, which binds retinoic acid to regulate immune recruitment, suggesting increased demand for inflammatory modulation (Núñez et al., 2010). The kidney and mesenteric LN in clinical disease demonstrated increased gene expression of *STRA6*, a membrane receptor for RBP to take up vitamin A from circulation (Kawaguchi et al., 2007). In the mesenteric LN, this may be support induction of gut trafficking receptors, α4β7 and CCR9 (Guo et al., 2014). The role of vitamin A in the kidney remains unclear; although significant irreversible loss through urinary excretion is considered unlikely (Gieng et al., 2007). However, due to the rapid degradation of retinol and sampling logistics, we were unable to measure retinol in feces or urine to confirm excretion due to disease.

**Figure 4.**
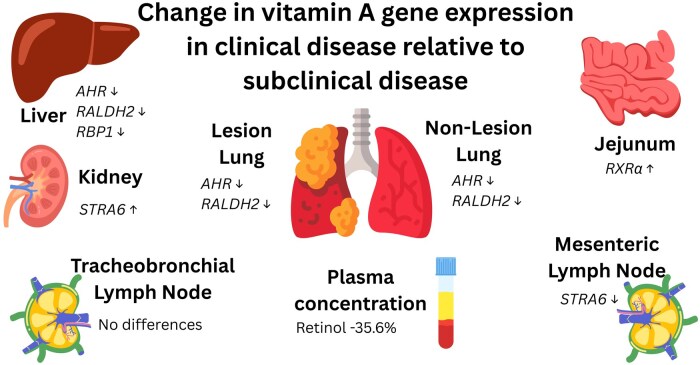
Change in gene expression in tissues from steers in clinical disease relative to subclinical disease. Steers were allocated into clinical and subclinical groups based on plasma Fe concentrations, lung pathology scores, and rectal temperatures during the five days prior to necropsy. All differences shown *P *≤ 0.05.

In the lung, NLL had increased gene expression of *RBP4*, *RALDH2*, and *RXRα*, consistent with greater vitamin A export (RBP4), activation (RALDH2), and signaling (RXRα) compared to LL. By contrast, LL expressed more *STRA6* and *MUC5AC*, suggesting greater demand for vitamin A from circulation (STRA6) and potential regulation of mucus production. While mucus aids pathogen clearance (Ehre et al., 2012) excessive production may contribute to lung congestion (Shao et al., 2004). Overall, tissue state (lesion compared to non-lesion) had a greater effect on vitamin A metabolism than disease severity, with demands changing as tissue became lesioned. A summary of vitamin A-related changes is shown in Figure 4.

In the lung, clinical disease increased Fe concentrations compared to subclinical disease and NLL had increased Fe concentrations compared to LL. Concentrations of Mn were only increased in LL compared to NLL. Although these minerals may utilize the same transporters such as divalent metal transporter 1 (Forbes and Gros, 2003) and ferroportin (Madejczyk and Ballatori, 2012), the affinity for Fe is greater than for Mn (Illing et al., 2012).

Plasma Fe was decreased in clinical disease, consistent with nutritional immunity, and this was accompanied by increased hepatic Fe and hepcidin expression. Despite these changes, there were no differences in the apparent absorption or retention of Fe and Mn. Tissue redistribution was influenced by disease state, however. Tracheobronchial LN had markedly higher Fe (64.5%) and Mn (20.9%) in clinical disease, suggesting increased demand for these micronutrients. A previous study has found correlations of micronutrient concentrations of LN with active germinal centers (Gorchakova et al., 2020), suggesting micronutrients may have a role in the proliferation of lymphoid cells in the LN. The pancreas of animals with clinical disease had 26.1% less Mn compared to subclinical disease. In the pancreas, Mn is utilized for the normal synthesis and secretion of insulin (Baly et al., 1984). The observed changes may reflect increased sequestration or altered insulin-related processes, though blood glucose and insulin were not measured in the present study.

In contrast to other trace minerals, plasma Cu concentrations increased by 28.8% during clinical disease, consistent with the increase in ceruloplasmin that occurs during the acute phase response. Despite this systemic increase, hepatic serum amyloid A 1 (SAA1) expression did not differ between groups. Copper concentrations were increased in LL of clinically diseased animals compared to NLL, although subclinical disease LL and NLL were not different from clinical disease LL and NLL. It is possible that there is greater influence on Cu concentrations during more severe disease, although overall tissue Cu concentrations were low with the exception of liver Cu in these beef on dairy steers. Concentrations of Cu were increased in the tracheobronchial LN and spleen of clinical disease. As peripheral lymphoid tissues are where naïve lymphocytes encounter antigens (Jenkins et al., 2001; Jung et al., 2010), increased Cu in these sites may support early immune responses.

Necropsy timing relative to stage of disease varied across animals, so some peaks or nadirs in rapidly changing responses, such as gene expression, may have been missed. Clinically ill animals showed greater variability in mineral metabolism and concentrations, though this must be interpreted in light of differences in disease stage and time of sampling. These findings highlight the need to consider disease state and timing of sampling when assessing micronutrient status. Animals sampled during illness may be misclassified as deficient or toxic, depending on the sample type and timing. Further research is needed to clarify micronutrient metabolism across different disease stages.

This study examined micronutrient absorption, retention tissue concentrations, and gene expression during a respiratory disease challenge. Although overall absorption and retention did not differ, disease severity altered systemic and local micronutrient metabolism. Redistribution of Zn and Cu without increased excretion highlights their importance during disease. Differential regulation of vitamin A metabolism reinforces its importance in infection responses, with potential roles in immune cell recruitment, barrier integrity, and inflammatory balance. These findings underscore the need to define micronutrient requirements of sick animals. Micronutrients show promise in supporting outcomes or reducing incidences of BRD; however, more work is needed to define their roles and optimize application in production.

## Supplementary Material

skaf453_Supplementary_Data

## Abbreviations:

AHR: aryl hydrocarbon receptor
; BHI: brain–heart infusion;
BRD: bovine respiratory disease;
BRSV: bovine respiratory syncytial virus
; BW: bodyweight;
cDNA: complementary deoxyribonucleic acid;
CFU: colony forming units;
CSHP: cottonseed hull pellet;
DART: depression appetite respiratory temperature;
DMI: dry matter intake;
ICP-OES: inductively coupled plasma optical emission spectrometry;
LL: lesion lung;
LN: lymph node
; LPS: lipopolysaccharide
; N: nitrogen
; NLL: non-lesion lung
; OM: organic matter
; PBS: phosphate buffered saline
; RALDH2: retinaldehyde dehydrogenase 2
; RBP: retinol binding protein
; RSV: respiratory syncytial virus
; RXR: retinoid X receptor
; SAA1: serum amyloid A1
; TCID_50_: tissue culture infectious dose
