# Supplementary material for: Impact of bovine respiratory disease on tissue-specific regulation of Zn and vitamin a metabolism and apparent absorption and retention of trace minerals
Source: J Anim Sci. 2025 Dec 31;104:skaf453. doi: 10.1093/jas/skaf453 (PMC12923155; doi:10.1093/jas/skaf453)

**Impact of bovine respiratory disease on tissue-specific regulation of Zn and vitamin A metabolism and apparent absorption and retention of trace minerals.**

Supplementary Materials

| **Supplementary Table 1**. Average dry matter percentage of tissues | | |
| --- | --- | --- |
|  | DM% | SEM |
| Thymus | 26.1 | 1.49 |
| Kidney | 19.3 | 0.47 |
| Pancreas | 23.7 | 0.40 |
| Spleen | 26.9 | 0.43 |
| Draining LN | 18.8 | 0.20 |
| Non-Lesion Lung | 16.9 | 2.55 |
| Lesion Lung | 15.8 | 0.58 |
| Liver | 27.6 | 0.37 |

| **Supplementary Table 2.** Primer sequences | | | | |
| --- | --- | --- | --- | --- |
| Gene^1^ | Forward | reverse | accession # | Reference |
| AHR | GTGCAGAAAACTGTCAAGCC | GCAACATCAAAGAAGCTCTTG | [XM_612996](http://www.ncbi.nlm.nih.gov/entrez/query.fcgi?cmd=search&db=nucleotide&doptcmdl=genbank&term=XM_612996) | (Girolami et al., 2011) |
| BCMO1 | GGCTTACATTCGGGGTGTGA | CGTCCTTCGGTCGATGATGT | NM_001024559.1 | (Wei et al., 2020) |
| HAMP | TCCTTGTCCTGCTCAGCCTG | CAGCAGAAGATGCAGATGGGAA | NC_037345.1 (45994911..45996358) | (Roperto et al., 2017) |
| MMP9 | GACCAGGACAAGCTCTACGG | CAGAAGCCCCACTTCTTGTC | NM_174744.2 | (Nuttinck et al., 2008) |
| MUC5AC | CAGTACAGAGTGCATGGGGA | TTCACAAACACCTCCCCACT | XM_015470102.1 | (Surlis et al., 2017) |
| OCLN | CTGCTGCCGACGAGTACAATAG | TTCCGTCGGTCGTAATCTCC | NM_001082433.2 | (Sacco et al., 2012) |
| RALDH2 | TCCCTGTCTGTAATCCAGCCAC | GAAAGCCAGCCTCCTTGATGAG |  | (Mohan et al., 2002) |
| RBP1 | CGGTCGACTTTACCGGGTACTG | GTCATGTCACTCATTCCTAGAGAC | NM_001025343 | (Lussier et al., 2017) |
| RBP4 | AGCTCCCGACAGGCGGACTC | AAGCTGCTCACCCGGCAGTC | NC_037353.1 (14896208..14902404, complement) | (Mullen et al., 2012) |
| RXRα | GTAACGTCCTTGCTGTGTTTG | GATTGGTCTACTGAAGGTCTGG | [XM_024998424.2](https://www.ncbi.nlm.nih.gov/entrez/viewer.fcgi?db=nucleotide&id=2587625541) |  |
| S100A8 | ATTTTGGGGAGACCTGGTGG | ACGGCGTGGTAATTCCCTTT | NM_002964.4 | (Park et al., 2016) |
| STRA6 | CTGTCTTCATGGTCCTCTTCA | GGTAATACAGCAGAGCCAGTATC | NC_037348.1 (34478073..34507705) |  |
| ZIP1 | TGCATGTGACGCTCCAGTTC | GTGGCCCACCATTCACTGTA | NM_00103581.2 | (Pascua et al., 2020) |
| ZIP2 | GTGCTCTCCATCCTGTCTTTAG | TCAGAGGGCGAAGTCATTTG | NC_037337.1:c26097864-26094678 | (Franco et al., 2024) |
| ZIP4 | CTCTTGCTGCCCCTGGAC | CCACCAGATCTGCGCGAG | [NC_037334.1:20936238–20946131](https://www.ncbi.nlm.nih.gov/nuccore/NC_037334.1) | (Franco et al., 2024) |
| ZIP8 | GGAGTGGAGGGAAGAAAGAAG | CTCACCTCGCCTGTGTATTT | NC_037333.1:22459057–22542658 | (Franco et al., 2024) |
| ZIP14 | AGGCTCCTGCTCTACTTC | AGCGTCTCAGAGGTATAATG | [NC_037335.1:69680220–69731758](https://www.ncbi.nlm.nih.gov/nuccore/NC_037335.1) | (Franco et al., 2024) |
| ZNT1 | GCAACTTGCTGGAAGCAGAA | TCAGGCTGAATGGTGGTAGC | NM_001205893.2 | (Ma et al., 2020) |
| ZNT4 | ATCACCATCCACAACATCCC | ATCCCAATTCCAAGGGCTAAA | NC_037337.1:64938017–64975659 | (Franco et al., 2024) |
| RPS9 | CGCCTCGACCAAGAGCTGAAG | CCTCCAGACCTCACGTTTGTTCC | NM_001101152.2 | (Sacco et al., 2012) |
| ^1^ AHR = aryl hydrocarbon receptor ; BCMO1 = beta-carotene 15,15' monooxygenase; HAMP = hepcidin; MMP9 = matrix metalloproteinase 9; MUC5AC = mucin 5 AC; OCLN = occludin; RALDH2 = retinaldehyde dehydrogenase 2; RBP = retinol binding protein; RXRα = retinoid X receptor α; S100A8 = S100 calcium binding protein A8; STRA6 = signaling receptor and transporter of retinol; ZIP = Zrt-, Irt-related protein; ZNT = Zn transporter; RPS9 = ribosomal protein S9 | | | | |

**Supplementary Figure 1.** Lungs were assigned a score based on the percentage of lung affected by gross pneumonic lesions. 6 = free of lesions; 5 = 1% to 5% affected; 4 = 6% to 15%; 3 = 16% to 30%; 2 = 31% to 50%; 1 = >50%. A. Lungs with a score of 1. B. Lungs with a score of 3. C. Lungs with a score of 5.
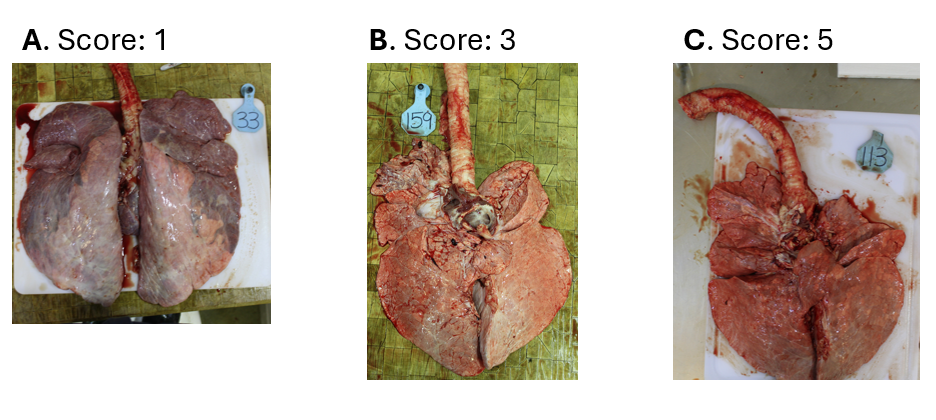

Supplement: skaf453_Supplementary_Data [file skaf453_supplementary_data.docx]
